# Thulium nickel/lithium distannide, TmNi_1−*x*_Li_*x*_Sn_2_ (*x* = 0.035)

**DOI:** 10.1107/S1600536813027335

**Published:** 2013-10-12

**Authors:** Andrij Stetskiv, Ivan Tarasiuk, Renata Misztal, Volodymyr Pavlyuk

**Affiliations:** a Ivano-Frankivsk National Medical University, Department of Chemistry, Galytska str. 2, 76018 Ivano-Frankivsk, Ukraine; b Department of Inorganic Chemistry, Ivan Franko Lviv National University, Kyryla and Mefodiya str. 6, 79005 Lviv, Ukraine; c Institute of Chemistry, Environment Protection and Biotechnology, Jan Dlugosz University, al. Armii Krajowej 13/15, 42-200 Czestochowa, Poland

## Abstract

The quaternary thulium nickel/lithium distannide, TmNi_1−*x*_Li_*x*_Sn_2_ (*x* = 0.035), crystallizes in the ortho­rhom­bic LuNiSn_2_ structure type. The asymmetric unit contains three Tm sites, six Sn sites, two Ni sites and one Ni/Li site [relative occupancies = 0.895 (8):0.185 (8)]. Site symmetries are .*m*. for all atoms. The 17-, 18- and 19-vertex distorted pseudo-Frank–Kasper polyhedra are typical for all Tm atoms. Four Sn atoms are enclosed in a 12-vertex deformed cubo­octa­hedron, and another Sn atom is enclosed in a penta­gonal prism with three added atoms. A tricapped trigonal prism is typical for a further Sn atom. The coordination number for all Ni atoms and Ni/Li statistical mixtures is 12 (fourcapped trigonal prism [Ni/LiTm_5_Sn_5_]). Tm atoms form the base of a prism and Ni/Li atoms are at the centres of the side faces of an [SnTm_6_Ni/Li_3_] prism. These isolated prisms are implemented into three-dimensional-nets built out of Sn atoms. Electronic structure calculations using TB-LMTO-ASA suggest that the Tm and Ni/Li atoms form positively charged *n*[TmNi/Li]^*m*+^ polycations which compensate the negative charge of 2*n*[Sn]^*m*−^ polyanions. Analysis of the inter­atomic distances and electronic structure calculations indicate the dominance of a metallic type of bonding.

## Related literature

For isotypic structures, see: Komarovskaya *et al.* (1983 ▶). For background of the study and related structures, see: Pavlyuk & Bodak (1992*a* ▶,*b* ▶); Pavlyuk *et al.* (1989*a* ▶,*b* ▶, 1991 ▶, 1993 ▶); Stetskiv *et al.* (2012 ▶, 2013 ▶). For electronic structure calculations, see: Andersen *et al.* (1986 ▶).

## Experimental

### 

#### Crystal data

TmNi_0.965_Li_0.035_Sn_2_
*M* *_r_* = 463.23
Orthorhombic, 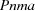

*a* = 16.0285 (11) Å
*b* = 4.3862 (4) Å
*c* = 14.3684 (10) Å
*V* = 1010.16 (14) Å^3^
*Z* = 12
Mo *K*α radiation
μ = 45.77 mm^−1^
*T* = 293 K
0.07 × 0.03 × 0.02 mm

#### Data collection

Oxford Diffraction Xcalibur3 CCD diffractometer
Absorption correction: analytical (*CrysAlis RED*; Oxford Diffraction, 2008 ▶) *T*_min_ = 0.213, *T*_max_ = 0.403
6737 measured reflections
1304 independent reflections
1096 reflections with *I* > 2σ(*I*)
*R*_int_ = 0.034

#### Refinement

*R*[*F* ^2^ > 2σ(*F* ^2^)] = 0.026
*wR*(*F* ^2^) = 0.060
*S* = 1.18
1304 reflections
76 parameters
Δρ_max_ = 2.07 e Å^−3^
Δρ_min_ = −2.13 e Å^−3^

### 

Data collection: *CrysAlis CCD* (Oxford Diffraction, 2008 ▶); cell refinement: *CrysAlis CCD*; data reduction: *CrysAlis RED* (Oxford Diffraction, 2008 ▶); program(s) used to solve structure: *SHELXS97* (Sheldrick, 2008 ▶); program(s) used to refine structure: *SHELXL97* (Sheldrick, 2008 ▶); molecular graphics: *DIAMOND* (Brandenburg, 2006 ▶); software used to prepare material for publication: *SHELXL97*.

## Supplementary Material

Crystal structure: contains datablock(s) I, New_Global_Publ_Block. DOI: 10.1107/S1600536813027335/ff2120sup1.cif

Structure factors: contains datablock(s) I. DOI: 10.1107/S1600536813027335/ff2120Isup2.hkl

Additional supplementary materials:  crystallographic information; 3D view; checkCIF report

## Figures and Tables

**Fig. 1.**
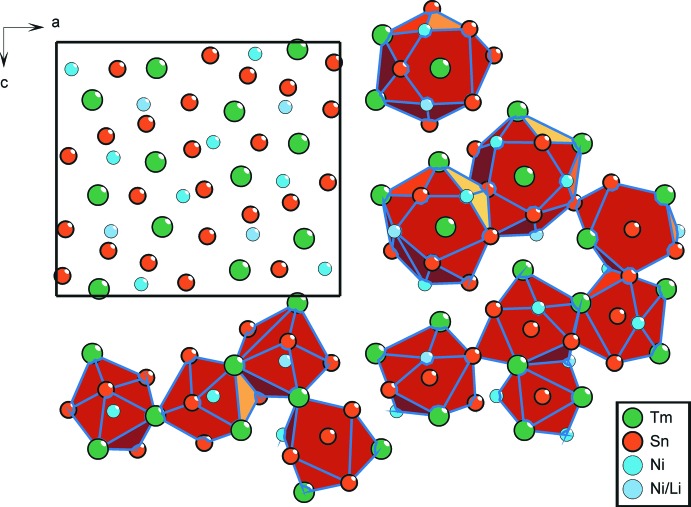
The projection of the unit cell and coordination polyhedra of the atoms.

**Fig. 2.**
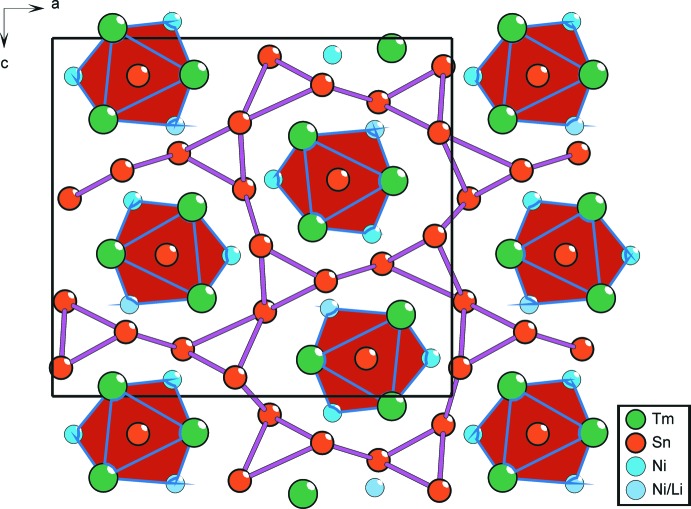
The distribution of thulium and nickel/lithium atoms in three-dimensional-nets built of the Sn atoms.

**Fig. 3.**
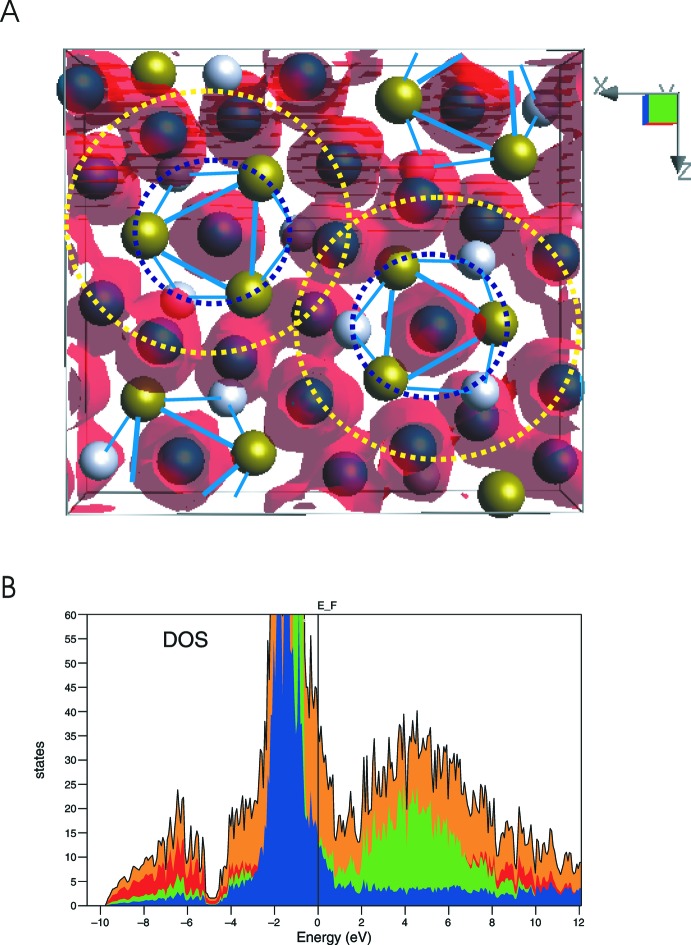
(A) Isosurfaces of electron localization function (ELF) around the atoms, positively charged n[TmNi/Li]m+ polycations marked by the violet dotted line and the negatively charged 2n[Sn]m- polyanions marked by the yellow dotted line. (B) Total and partial DOS.

## supplementary crystallographic information

### 1. Comment

The RETSn_2_ and RET*_x_*Sn2 (*x*<1) type metallic compounds where RE is a rare-earth element (Gd—Lu) and T is a d-electron element crystallize in different orthorhombic crystal structures LuNiSn_2_ (space group *Pnma*) and CeNiSi_2_-type (space group *Cmcm*) respectively. In the ternary RELiSn_2_ compounds lithium atoms occupy the same crystallographic position as the atoms of transition metal in the original CeNiSi_2_ structure type (Pavlyuk *et al.*, 1989*a*). Previous structural studies of the four-component alloys from TbLiSn_2_–TbZnSn_2_ sections indicate the existence of TbLi_1–_*_x_*Zn*_x_*Sn_2_ limited solid solution (Stetskiv *et al.*, 2012). X-ray single-crystal study showed that the TbLi_1–_*_x_*Zn*_x_*Sn_2_ solid solution was formed by the partial substitution of lithium atoms by zinc atoms in *4c* site. The ability of lithium atoms to partially substitute the atoms of transition metals was previously observed by us while studying solid solutions RELi*_x_*Cu_2–_*_x_*Si_2_ and RELi*_x_*Cu_2–_*_x_*Ge_2_ (Pavlyuk *et al.*, 1993). The ordered substitution of transition metals by lithium is observed for Tm_2.22_Co_6_Sn_20_ and TmLi_2_Co_6_Sn_20_ stannides (Stetskiv *et al.*, 2013). The ability of lithium atoms to occupy the same crystallographic position as the atoms of transition metal was observed previously while studying compounds RELiGe with the ZrNiAl-type (Pavlyuk *et al.*, 1991 and Pavlyuk & Bodak, 1992*a*), RE_3_Li_2_Ge_3_ with Hf_3_Ni_2_Si_3_-type (Pavlyuk & Bodak, 1992*b*) and Yb_5_Li_4_Ge_4_ with Nb_5_Cu_4_Si_4_-type (Pavlyuk *et al.*, 1989*b*).

The four-component phase TmNi_1–x_Li*_x_*Sn_2_ with low content of lithium from the TmLiSn_2_–TmNiSn_2_ section was detected by us during the systematic study of alloys of Tm—Ni—Li—Sn system. Selected single-crystal data show that the title compound crystallizes with the orthorhombic space group *Pnma* as a LuNiSn_2_-type (Komarovskaya *et al.*, 1983). The projection of the unit cell and coordination polyhedra of the atoms are shown in Fig. 1. The Tm atoms are enclosed in 17-, 18- and 19-vertex distorted pseudo Frank-Kasper polyhedra. The coordination polyhedron of Sn4, Sn7, Sn8 and Sn9 atoms is 12-vertex distorted cubooctahedron. The Sn5 is enclosed in pentagonal prism with three added atoms. The tricapped trigonal prism is typical for Sn6 atom. The environment of the Ni atoms and Ni/Li statistical mixture is a fourcapped trigonal prism and a coordination number equals 10 (Tm_5_Sn_5_).

The distribution of nickel/lithium and thulium atoms in three-dimensional-nets built of Sn atoms is shown in Fig. 2. The thulium and nickel/lithium atoms form tricapped trigonal prism around Sn6. Thulium atoms form the base of prism and nickel/lithium atoms centre side faces of [SnTm_6_Ni/Li_3_] prism. These isolated prisms are implemented into three-dimensional-nets built of tin atoms. The data of electronic structure calculations using the TB-LMTO-ASA (Andersen *et al.*, 1986) suggest that thulium and nickel/lithium atoms form a positively charged *n[TmNi/Li]^m+^* polycations which compensate the negative charge of *2n[Sn]^m-^* polyanions (Fig. 3 A). Of course, this suggestion is based on the partial charges. All interatomic distances are values which correlate well with the atomic size; metallic type of bonding was indicated. A significant density of states (DOS) at the Fermi level (Fig. 3B) also indicates dominance of metallic bonding.

### 2. Experimental

Thulium, nickel, lithium and tin, all with a nominal purity more than 99.9 wt. %, were used as starting elements. First, the pieces of the pure metals with a stoichiometry Tm_25_Ni_20_Li_5_Sn_50_ were pressed into pellet, enclosed in tantalum crucible and placed in a resistance furnace with a thermocouple controller. Heating rate from room temperature to 670 K was equal to 5 K per minute. At this temperature the alloy was kept over 2 d and then the temperature was increased from 670 to 1070 K over 1 h. Then, the alloy was annealed at this temperature for 8 h and slowly cooled down to room temperature. After the melting and annealing procedures, the total weight loss was less than 2%. Small good quality single-crystal of the title compound was isolated from the alloy.

The synthesized alloy is practically single-phase. Therefore, in order to confirm the accuracy of the compositions, the density of the alloy was determined using the volumetric method. The measured density is 9.08 (5) Mg m^-3^, and these values differ by less than 1% from the densities calculated from the X-ray data. For the TmNiSn_2_ ternary phase density is 9,19 Mg m^-3^ (Komarovskaya *et al.*, 1983).

### 3. Refinement

The structure of the title phase was solved by direct methods after the analytical absorption correction. In the first stage of the refinement, the thermal displacement parameter of Ni12 atom was considerably different from those of other Ni sites, suggesting that this position is partially occupied by the lithium atom. In the final refinement cycles all atoms were successfully refined with anisotropic displacement parameters.

### Crystal data

**Table:**

TmNi_0.965_Li_0.035_Sn_2_	*F*(000) = 2353.5
*M**_r_* = 463.23	*D*_x_ = 9.138 Mg m^−^^3^
Orthorhombic, *P**n**m**a*	Mo *K*α radiation, λ = 0.71073 Å
Hall symbol: -P 2ac 2n	Cell parameters from 1304 reflections
*a* = 16.0285 (11) Å	θ = 3.8–27.5°
*b* = 4.3862 (4) Å	µ = 45.77 mm^−^^1^
*c* = 14.3684 (10) Å	*T* = 293 K
*V* = 1010.16 (14) Å^3^	Prism, metallic dark gray
*Z* = 12	0.07 × 0.03 × 0.02 mm

### Data collection

**Table:**

Oxford Diffraction Xcalibur3 CCD diffractometer	1304 independent reflections
Radiation source: fine-focus sealed tube	1096 reflections with *I* > 2σ(*I*)
Graphite monochromator	*R*_int_ = 0.034
Detector resolution: 0 pixels mm^-1^	θ_max_ = 27.5°, θ_min_ = 3.8°
ω scans	*h* = −20→20
Absorption correction: analytical (*CrysAlis RED*; Oxford Diffraction, 2008)	*k* = −3→5
*T*_min_ = 0.213, *T*_max_ = 0.403	*l* = −18→18
6737 measured reflections	

### Refinement

**Table:**

Refinement on *F*^2^	Primary atom site location: structure-invariant direct methods
Least-squares matrix: full	Secondary atom site location: difference Fourier map
*R*[*F*^2^ > 2σ(*F*^2^)] = 0.026	*w* = 1/[σ^2^(*F*_o_^2^) + (0.023*P*)^2^ + 11.3465*P*] where *P* = (*F*_o_^2^ + 2*F*_c_^2^)/3
*wR*(*F*^2^) = 0.060	(Δ/σ)_max_ = 0.006
*S* = 1.18	Δρ_max_ = 2.07 e Å^−^^3^
1304 reflections	Δρ_min_ = −2.13 e Å^−^^3^
76 parameters	Extinction correction: *SHELXL97* (Sheldrick, 2008), Fc^*^=kFc[1+0.001xFc^2^λ^3^/sin(2θ)]^-1/4^
0 restraints	Extinction coefficient: 0.00030 (3)

### Special details

**Table:**

Geometry. All e.s.d.'s (except the e.s.d. in the dihedral angle between two l.s. planes) are estimated using the full covariance matrix. The cell e.s.d.'s are taken into account individually in the estimation of e.s.d.'s in distances, angles and torsion angles; correlations between e.s.d.'s in cell parameters are only used when they are defined by crystal symmetry. An approximate (isotropic) treatment of cell e.s.d.'s is used for estimating e.s.d.'s involving l.s. planes.
Refinement. Refinement of *F*^2^ against ALL reflections. The weighted *R*-factor *wR* and goodness of fit *S* are based on *F*^2^, conventional *R*-factors *R* are based on *F*, with *F* set to zero for negative *F*^2^. The threshold expression of *F*^2^ > σ(*F*^2^) is used only for calculating *R*-factors(gt) *etc*. and is not relevant to the choice of reflections for refinement. *R*-factors based on *F*^2^ are statistically about twice as large as those based on *F*, and *R*- factors based on ALL data will be even larger.

### Fractional atomic coordinates and isotropic or equivalent isotropic displacement parameters (Å^2^)

**Table:**

	*x*	*y*	*z*	*U*_iso_*/*U*_eq_	Occ. (<1)
Tm1	0.35386 (4)	0.2500	0.10177 (4)	0.01166 (15)	
Tm2	0.15061 (4)	0.2500	−0.02797 (4)	0.01120 (15)	
Tm3	0.12704 (4)	0.2500	0.22713 (4)	0.01269 (15)	
Sn4	0.02119 (5)	−0.2500	−0.07931 (6)	0.0105 (2)	
Sn5	0.46864 (6)	−0.2500	0.23607 (7)	0.0158 (2)	
Sn6	0.21452 (6)	−0.2500	0.10507 (6)	0.0129 (2)	
Sn7	0.32559 (6)	0.2500	−0.13071 (6)	0.0118 (2)	
Sn8	0.31632 (6)	0.2500	0.32114 (7)	0.0157 (2)	
Sn9	0.45722 (6)	−0.2500	−0.05229 (7)	0.0154 (2)	
Ni10	0.29945 (11)	−0.2500	−0.04622 (13)	0.0133 (4)	
Ni11	0.05291 (10)	−0.2500	0.10639 (12)	0.0122 (4)	
Ni12	0.30709 (12)	−0.2500	0.24440 (14)	0.0162 (7)	0.895 (8)
Li12	0.30709 (12)	−0.2500	0.24440 (14)	0.0162 (7)	0.105 (8)

### Atomic displacement parameters (Å^2^)

**Table:**

	*U*^11^	*U*^22^	*U*^33^	*U*^12^	*U*^13^	*U*^23^
Tm1	0.0109 (3)	0.0140 (3)	0.0100 (3)	0.000	0.0005 (2)	0.000
Tm2	0.0107 (3)	0.0124 (3)	0.0105 (3)	0.000	0.0011 (2)	0.000
Tm3	0.0157 (3)	0.0136 (3)	0.0087 (3)	0.000	−0.0028 (2)	0.000
Sn4	0.0092 (4)	0.0106 (4)	0.0117 (4)	0.000	0.0002 (3)	0.000
Sn5	0.0162 (5)	0.0174 (4)	0.0138 (5)	0.000	−0.0035 (4)	0.000
Sn6	0.0117 (5)	0.0124 (4)	0.0145 (5)	0.000	0.0009 (3)	0.000
Sn7	0.0140 (5)	0.0106 (4)	0.0107 (5)	0.000	0.0014 (3)	0.000
Sn8	0.0114 (5)	0.0231 (5)	0.0127 (5)	0.000	−0.0001 (3)	0.000
Sn9	0.0087 (5)	0.0174 (5)	0.0201 (5)	0.000	−0.0015 (4)	0.000
Ni10	0.0117 (9)	0.0143 (8)	0.0138 (9)	0.000	−0.0009 (7)	0.000
Ni11	0.0116 (8)	0.0124 (8)	0.0127 (9)	0.000	−0.0004 (6)	0.000
Ni12	0.0220 (12)	0.0148 (11)	0.0117 (11)	0.000	−0.0044 (8)	0.000
Li12	0.0220 (12)	0.0148 (11)	0.0117 (11)	0.000	−0.0044 (8)	0.000

### Geometric parameters (Å, º)

**Table:**

Tm1—Li12^i^	3.0938 (15)	Sn5—Tm1^xi^	3.4522 (9)
Tm1—Ni12^i^	3.0938 (15)	Sn6—Ni12	2.492 (2)
Tm1—Ni12	3.0938 (15)	Sn6—Ni10	2.565 (2)
Tm1—Sn9^ii^	3.1104 (11)	Sn6—Ni11	2.5904 (19)
Tm1—Sn6	3.1306 (8)	Sn6—Tm2^xi^	3.0844 (8)
Tm1—Sn6^i^	3.1306 (8)	Sn6—Tm1^xi^	3.1306 (8)
Tm1—Ni10	3.1768 (14)	Sn6—Tm3^xi^	3.1387 (8)
Tm1—Ni10^i^	3.1768 (14)	Sn7—Ni10^i^	2.5415 (10)
Tm1—Sn8	3.2089 (11)	Sn7—Ni10	2.5415 (10)
Tm1—Sn7	3.3711 (11)	Sn7—Li12^iii^	2.783 (2)
Tm1—Sn5^i^	3.4522 (9)	Sn7—Ni12^iii^	2.783 (2)
Tm2—Sn6^i^	3.0844 (8)	Sn7—Tm3^iii^	3.0916 (8)
Tm2—Sn6	3.0844 (8)	Sn7—Tm3^iv^	3.0916 (8)
Tm2—Sn4	3.1076 (8)	Sn7—Sn8^iii^	3.2345 (9)
Tm2—Sn4^i^	3.1076 (8)	Sn7—Sn8^iv^	3.2345 (9)
Tm2—Sn8^iii^	3.1290 (8)	Sn7—Sn9^i^	3.2451 (10)
Tm2—Sn8^iv^	3.1290 (8)	Sn7—Sn9	3.2451 (10)
Tm2—Sn4^v^	3.1557 (11)	Sn8—Ni12	2.4591 (10)
Tm2—Sn7	3.1695 (11)	Sn8—Li12^i^	2.4591 (10)
Tm2—Ni10^i^	3.2512 (13)	Sn8—Ni12^i^	2.4591 (10)
Tm2—Ni10	3.2512 (13)	Sn8—Ni10^vii^	2.660 (2)
Tm2—Ni11^i^	3.3150 (13)	Sn8—Sn4^vii^	2.9714 (13)
Tm2—Ni11	3.3150 (13)	Sn8—Tm2^vi^	3.1290 (8)
Tm3—Ni11	3.0383 (13)	Sn8—Tm2^vii^	3.1290 (8)
Tm3—Ni11^i^	3.0383 (13)	Sn8—Sn7^vii^	3.2345 (9)
Tm3—Sn7^vi^	3.0916 (8)	Sn8—Sn7^vi^	3.2345 (9)
Tm3—Sn7^vii^	3.0916 (8)	Sn9—Ni10	2.5303 (19)
Tm3—Sn6	3.1387 (8)	Sn9—Sn9^ii^	2.9914 (13)
Tm3—Sn6^i^	3.1387 (8)	Sn9—Sn9^xiv^	2.9914 (13)
Tm3—Sn4^v^	3.1868 (10)	Sn9—Tm1^ii^	3.1104 (11)
Tm3—Sn8	3.3210 (11)	Sn9—Sn7^xi^	3.2451 (10)
Tm3—Sn5^viii^	3.3963 (8)	Sn9—Tm3^iii^	3.4450 (12)
Tm3—Sn5^ix^	3.3963 (8)	Sn9—Tm1^xi^	3.5291 (9)
Tm3—Sn9^vii^	3.4450 (12)	Ni10—Sn7^xi^	2.5415 (10)
Tm3—Ni10^vii^	3.4631 (19)	Ni10—Sn8^iii^	2.660 (2)
Sn4—Ni11^x^	2.5242 (9)	Ni10—Tm1^xi^	3.1768 (14)
Sn4—Ni11^v^	2.5242 (9)	Ni10—Tm2^xi^	3.2512 (13)
Sn4—Ni11	2.7163 (19)	Ni10—Tm3^iii^	3.4631 (19)
Sn4—Sn8^iii^	2.9714 (13)	Ni11—Sn4^x^	2.5242 (9)
Sn4—Tm2^xi^	3.1076 (8)	Ni11—Sn4^v^	2.5242 (9)
Sn4—Tm2^v^	3.1557 (11)	Ni11—Sn5^viii^	2.636 (2)
Sn4—Tm3^v^	3.1868 (10)	Ni11—Tm3^xi^	3.0383 (13)
Sn4—Sn4^v^	3.2350 (13)	Ni11—Tm2^xi^	3.3150 (13)
Sn4—Sn4^x^	3.2350 (13)	Ni11—Tm2^v^	3.4512 (17)
Sn5—Ni12	2.592 (2)	Ni12—Sn8^xi^	2.4591 (10)
Sn5—Ni11^xii^	2.636 (2)	Ni12—Sn7^vii^	2.783 (2)
Sn5—Tm3^xii^	3.3963 (9)	Ni12—Tm1^xi^	3.0938 (15)
Sn5—Tm3^xiii^	3.3963 (8)	Ni12—Tm2^vii^	3.340 (2)
			
Li12^i^—Tm1—Ni12^i^	0.00 (9)	Tm2—Sn6—Tm3	72.611 (17)
Li12^i^—Tm1—Ni12	90.29 (6)	Tm1—Sn6—Tm3	80.659 (16)
Ni12^i^—Tm1—Ni12	90.29 (6)	Tm1^xi^—Sn6—Tm3	144.75 (4)
Li12^i^—Tm1—Sn9^ii^	112.79 (4)	Tm3^xi^—Sn6—Tm3	88.65 (3)
Ni12^i^—Tm1—Sn9^ii^	112.79 (4)	Ni10^i^—Sn7—Ni10	119.29 (8)
Ni12—Tm1—Sn9^ii^	112.79 (4)	Ni10^i^—Sn7—Li12^iii^	100.49 (5)
Li12^i^—Tm1—Sn6	108.28 (4)	Ni10—Sn7—Li12^iii^	100.49 (5)
Ni12^i^—Tm1—Sn6	108.28 (4)	Ni10^i^—Sn7—Ni12^iii^	100.49 (5)
Ni12—Tm1—Sn6	47.19 (4)	Ni10—Sn7—Ni12^iii^	100.49 (5)
Sn9^ii^—Tm1—Sn6	134.268 (15)	Li12^iii^—Sn7—Ni12^iii^	0.00 (8)
Li12^i^—Tm1—Sn6^i^	47.19 (4)	Ni10^i^—Sn7—Tm3^iii^	165.51 (5)
Ni12^i^—Tm1—Sn6^i^	47.19 (4)	Ni10—Sn7—Tm3^iii^	75.16 (4)
Ni12—Tm1—Sn6^i^	108.28 (4)	Li12^iii^—Sn7—Tm3^iii^	76.21 (4)
Sn9^ii^—Tm1—Sn6^i^	134.268 (15)	Ni12^iii^—Sn7—Tm3^iii^	76.21 (4)
Sn6—Tm1—Sn6^i^	88.94 (3)	Ni10^i^—Sn7—Tm3^iv^	75.16 (4)
Li12^i^—Tm1—Ni10	150.02 (5)	Ni10—Sn7—Tm3^iv^	165.51 (5)
Ni12^i^—Tm1—Ni10	150.02 (5)	Li12^iii^—Sn7—Tm3^iv^	76.21 (4)
Ni12—Tm1—Ni10	83.54 (4)	Ni12^iii^—Sn7—Tm3^iv^	76.21 (4)
Sn9^ii^—Tm1—Ni10	96.56 (4)	Tm3^iii^—Sn7—Tm3^iv^	90.37 (3)
Sn6—Tm1—Ni10	47.98 (4)	Ni10^i^—Sn7—Tm2	68.39 (4)
Sn6^i^—Tm1—Ni10	107.33 (3)	Ni10—Sn7—Tm2	68.39 (4)
Li12^i^—Tm1—Ni10^i^	83.54 (4)	Li12^iii^—Sn7—Tm2	67.91 (5)
Ni12^i^—Tm1—Ni10^i^	83.54 (4)	Ni12^iii^—Sn7—Tm2	67.91 (5)
Ni12—Tm1—Ni10^i^	150.02 (5)	Tm3^iii^—Sn7—Tm2	121.67 (2)
Sn9^ii^—Tm1—Ni10^i^	96.56 (4)	Tm3^iv^—Sn7—Tm2	121.67 (2)
Sn6—Tm1—Ni10^i^	107.33 (3)	Ni10^i^—Sn7—Sn8^iii^	124.84 (5)
Sn6^i^—Tm1—Ni10^i^	47.98 (4)	Ni10—Sn7—Sn8^iii^	53.21 (4)
Ni10—Tm1—Ni10^i^	87.31 (5)	Li12^iii^—Sn7—Sn8^iii^	47.52 (2)
Li12^i^—Tm1—Sn8	45.89 (3)	Ni12^iii^—Sn7—Sn8^iii^	47.52 (2)
Ni12^i^—Tm1—Sn8	45.89 (3)	Tm3^iii^—Sn7—Sn8^iii^	63.28 (2)
Ni12—Tm1—Sn8	45.89 (3)	Tm3^iv^—Sn7—Sn8^iii^	120.82 (3)
Sn9^ii^—Tm1—Sn8	114.02 (3)	Tm2—Sn7—Sn8^iii^	58.49 (2)
Sn6—Tm1—Sn8	81.45 (2)	Ni10^i^—Sn7—Sn8^iv^	53.21 (4)
Sn6^i^—Tm1—Sn8	81.45 (2)	Ni10—Sn7—Sn8^iv^	124.84 (5)
Ni10—Tm1—Sn8	127.30 (3)	Li12^iii^—Sn7—Sn8^iv^	47.52 (2)
Ni10^i^—Tm1—Sn8	127.30 (3)	Ni12^iii^—Sn7—Sn8^iv^	47.52 (2)
Li12^i^—Tm1—Sn7	128.59 (3)	Tm3^iii^—Sn7—Sn8^iv^	120.82 (3)
Ni12^i^—Tm1—Sn7	128.59 (3)	Tm3^iv^—Sn7—Sn8^iv^	63.28 (2)
Ni12—Tm1—Sn7	128.59 (3)	Tm2—Sn7—Sn8^iv^	58.49 (2)
Sn9^ii^—Tm1—Sn7	84.51 (3)	Sn8^iii^—Sn7—Sn8^iv^	85.38 (3)
Sn6—Tm1—Sn7	85.36 (2)	Ni10^i^—Sn7—Sn9^i^	50.07 (4)
Sn6^i^—Tm1—Sn7	85.36 (2)	Ni10—Sn7—Sn9^i^	121.64 (5)
Ni10—Tm1—Sn7	45.56 (3)	Li12^iii^—Sn7—Sn9^i^	136.12 (2)
Ni10^i^—Tm1—Sn7	45.56 (3)	Ni12^iii^—Sn7—Sn9^i^	136.12 (2)
Sn8—Tm1—Sn7	161.47 (3)	Tm3^iii^—Sn7—Sn9^i^	123.31 (3)
Li12^i^—Tm1—Sn5^i^	46.26 (4)	Tm3^iv^—Sn7—Sn9^i^	65.81 (2)
Ni12^i^—Tm1—Sn5^i^	46.26 (4)	Tm2—Sn7—Sn9^i^	114.43 (3)
Ni12—Tm1—Sn5^i^	102.08 (4)	Sn8^iii^—Sn7—Sn9^i^	171.77 (4)
Sn9^ii^—Tm1—Sn5^i^	66.98 (2)	Sn8^iv^—Sn7—Sn9^i^	94.201 (17)
Sn6—Tm1—Sn5^i^	144.76 (3)	Ni10^i^—Sn7—Sn9	121.64 (5)
Sn6^i^—Tm1—Sn5^i^	85.797 (19)	Ni10—Sn7—Sn9	50.07 (4)
Ni10—Tm1—Sn5^i^	163.54 (4)	Li12^iii^—Sn7—Sn9	136.12 (2)
Ni10^i^—Tm1—Sn5^i^	94.70 (3)	Ni12^iii^—Sn7—Sn9	136.12 (2)
Sn8—Tm1—Sn5^i^	63.31 (2)	Tm3^iii^—Sn7—Sn9	65.81 (2)
Sn7—Tm1—Sn5^i^	128.72 (2)	Tm3^iv^—Sn7—Sn9	123.31 (3)
Sn6^i^—Tm2—Sn6	90.64 (3)	Tm2—Sn7—Sn9	114.43 (3)
Sn6^i^—Tm2—Sn4	150.53 (3)	Sn8^iii^—Sn7—Sn9	94.201 (17)
Sn6—Tm2—Sn4	82.359 (19)	Sn8^iv^—Sn7—Sn9	171.77 (4)
Sn6^i^—Tm2—Sn4^i^	82.359 (19)	Sn9^i^—Sn7—Sn9	85.04 (3)
Sn6—Tm2—Sn4^i^	150.53 (3)	Ni10^i^—Sn7—Tm1	63.18 (4)
Sn4—Tm2—Sn4^i^	89.78 (3)	Ni10—Sn7—Tm1	63.18 (4)
Sn6^i^—Tm2—Sn8^iii^	150.63 (3)	Li12^iii^—Sn7—Tm1	137.88 (5)
Sn6—Tm2—Sn8^iii^	82.81 (2)	Ni12^iii^—Sn7—Tm1	137.88 (5)
Sn4—Tm2—Sn8^iii^	56.91 (2)	Tm3^iii^—Sn7—Tm1	128.44 (2)
Sn4^i^—Tm2—Sn8^iii^	116.31 (3)	Tm3^iv^—Sn7—Tm1	128.44 (2)
Sn6^i^—Tm2—Sn8^iv^	82.81 (2)	Tm2—Sn7—Tm1	69.97 (2)
Sn6—Tm2—Sn8^iv^	150.63 (3)	Sn8^iii^—Sn7—Tm1	107.85 (3)
Sn4—Tm2—Sn8^iv^	116.31 (3)	Sn8^iv^—Sn7—Tm1	107.85 (3)
Sn4^i^—Tm2—Sn8^iv^	56.91 (2)	Sn9^i^—Sn7—Tm1	64.44 (2)
Sn8^iii^—Tm2—Sn8^iv^	89.00 (3)	Sn9—Sn7—Tm1	64.44 (2)
Sn6^i^—Tm2—Sn4^v^	89.26 (2)	Ni12—Sn8—Li12^i^	126.20 (9)
Sn6—Tm2—Sn4^v^	89.26 (2)	Ni12—Sn8—Ni12^i^	126.20 (9)
Sn4—Tm2—Sn4^v^	62.19 (2)	Li12^i^—Sn8—Ni12^i^	0.00 (12)
Sn4^i^—Tm2—Sn4^v^	62.19 (2)	Ni12—Sn8—Ni10^vii^	106.22 (5)
Sn8^iii^—Tm2—Sn4^v^	119.10 (2)	Li12^i^—Sn8—Ni10^vii^	106.22 (5)
Sn8^iv^—Tm2—Sn4^v^	119.10 (2)	Ni12^i^—Sn8—Ni10^vii^	106.22 (5)
Sn6^i^—Tm2—Sn7	89.70 (2)	Ni12—Sn8—Sn4^vii^	105.58 (5)
Sn6—Tm2—Sn7	89.70 (2)	Li12^i^—Sn8—Sn4^vii^	105.58 (5)
Sn4—Tm2—Sn7	118.70 (2)	Ni12^i^—Sn8—Sn4^vii^	105.58 (5)
Sn4^i^—Tm2—Sn7	118.70 (2)	Ni10^vii^—Sn8—Sn4^vii^	105.46 (5)
Sn8^iii^—Tm2—Sn7	61.80 (2)	Ni12—Sn8—Tm2^vi^	161.07 (5)
Sn8^iv^—Tm2—Sn7	61.80 (2)	Li12^i^—Sn8—Tm2^vi^	72.29 (4)
Sn4^v^—Tm2—Sn7	178.52 (3)	Ni12^i^—Sn8—Tm2^vi^	72.29 (4)
Sn6^i^—Tm2—Ni10^i^	47.67 (3)	Ni10^vii^—Sn8—Tm2^vi^	67.78 (3)
Sn6—Tm2—Ni10^i^	106.61 (3)	Sn4^vii^—Sn8—Tm2^vi^	61.18 (2)
Sn4—Tm2—Ni10^i^	161.22 (4)	Ni12—Sn8—Tm2^vii^	72.29 (4)
Sn4^i^—Tm2—Ni10^i^	89.69 (3)	Li12^i^—Sn8—Tm2^vii^	161.07 (5)
Sn8^iii^—Tm2—Ni10^i^	107.01 (3)	Ni12^i^—Sn8—Tm2^vii^	161.07 (5)
Sn8^iv^—Tm2—Ni10^i^	49.23 (3)	Ni10^vii^—Sn8—Tm2^vii^	67.78 (3)
Sn4^v^—Tm2—Ni10^i^	132.82 (3)	Sn4^vii^—Sn8—Tm2^vii^	61.18 (2)
Sn7—Tm2—Ni10^i^	46.61 (3)	Tm2^vi^—Sn8—Tm2^vii^	89.00 (3)
Sn6^i^—Tm2—Ni10	106.61 (3)	Ni12—Sn8—Tm1	64.59 (5)
Sn6—Tm2—Ni10	47.67 (3)	Li12^i^—Sn8—Tm1	64.59 (5)
Sn4—Tm2—Ni10	89.69 (3)	Ni12^i^—Sn8—Tm1	64.59 (5)
Sn4^i^—Tm2—Ni10	161.22 (4)	Ni10^vii^—Sn8—Tm1	146.57 (5)
Sn8^iii^—Tm2—Ni10	49.23 (3)	Sn4^vii^—Sn8—Tm1	107.97 (3)
Sn8^iv^—Tm2—Ni10	107.01 (3)	Tm2^vi^—Sn8—Tm1	130.45 (2)
Sn4^v^—Tm2—Ni10	132.82 (3)	Tm2^vii^—Sn8—Tm1	130.45 (2)
Sn7—Tm2—Ni10	46.61 (3)	Ni12—Sn8—Sn7^vii^	56.56 (5)
Ni10^i^—Tm2—Ni10	84.84 (4)	Li12^i^—Sn8—Sn7^vii^	131.17 (6)
Sn6^i^—Tm2—Ni11^i^	47.59 (3)	Ni12^i^—Sn8—Sn7^vii^	131.17 (6)
Sn6—Tm2—Ni11^i^	105.45 (3)	Ni10^vii^—Sn8—Sn7^vii^	49.92 (3)
Sn4—Tm2—Ni11^i^	106.85 (3)	Sn4^vii^—Sn8—Sn7^vii^	120.89 (3)
Sn4^i^—Tm2—Ni11^i^	49.91 (3)	Tm2^vi^—Sn8—Sn7^vii^	116.50 (3)
Sn8^iii^—Tm2—Ni11^i^	161.30 (4)	Tm2^vii^—Sn8—Sn7^vii^	59.72 (2)
Sn8^iv^—Tm2—Ni11^i^	91.14 (3)	Tm1—Sn8—Sn7^vii^	110.00 (3)
Sn4^v^—Tm2—Ni11^i^	45.84 (2)	Ni12—Sn8—Sn7^vi^	131.17 (6)
Sn7—Tm2—Ni11^i^	133.57 (3)	Li12^i^—Sn8—Sn7^vi^	56.56 (5)
Ni10^i^—Tm2—Ni11^i^	86.98 (3)	Ni12^i^—Sn8—Sn7^vi^	56.56 (5)
Ni10—Tm2—Ni11^i^	147.13 (5)	Ni10^vii^—Sn8—Sn7^vi^	49.92 (3)
Sn6^i^—Tm2—Ni11	105.45 (3)	Sn4^vii^—Sn8—Sn7^vi^	120.89 (3)
Sn6—Tm2—Ni11	47.59 (3)	Tm2^vi^—Sn8—Sn7^vi^	59.72 (2)
Sn4—Tm2—Ni11	49.91 (3)	Tm2^vii^—Sn8—Sn7^vi^	116.50 (3)
Sn4^i^—Tm2—Ni11	106.85 (3)	Tm1—Sn8—Sn7^vi^	110.00 (3)
Sn8^iii^—Tm2—Ni11	91.14 (3)	Sn7^vii^—Sn8—Sn7^vi^	85.38 (3)
Sn8^iv^—Tm2—Ni11	161.30 (4)	Ni12—Sn8—Tm3	76.27 (5)
Sn4^v^—Tm2—Ni11	45.84 (2)	Li12^i^—Sn8—Tm3	76.27 (5)
Sn7—Tm2—Ni11	133.57 (3)	Ni12^i^—Sn8—Tm3	76.27 (5)
Ni10^i^—Tm2—Ni11	147.13 (5)	Ni10^vii^—Sn8—Tm3	69.76 (5)
Ni10—Tm2—Ni11	86.98 (3)	Sn4^vii^—Sn8—Tm3	175.22 (4)
Ni11^i^—Tm2—Ni11	82.84 (4)	Tm2^vi^—Sn8—Tm3	115.88 (2)
Ni11—Tm3—Ni11^i^	92.41 (5)	Tm2^vii^—Sn8—Tm3	115.88 (2)
Ni11—Tm3—Sn7^vi^	170.17 (4)	Tm1—Sn8—Tm3	76.81 (3)
Ni11^i^—Tm3—Sn7^vi^	87.78 (3)	Sn7^vii^—Sn8—Tm3	56.26 (2)
Ni11—Tm3—Sn7^vii^	87.78 (3)	Sn7^vi^—Sn8—Tm3	56.26 (2)
Ni11^i^—Tm3—Sn7^vii^	170.17 (4)	Ni10—Sn9—Sn9^ii^	116.16 (5)
Sn7^vi^—Tm3—Sn7^vii^	90.37 (3)	Ni10—Sn9—Sn9^xiv^	116.16 (5)
Ni11—Tm3—Sn6	49.56 (3)	Sn9^ii^—Sn9—Sn9^xiv^	94.30 (5)
Ni11^i^—Tm3—Sn6	111.10 (4)	Ni10—Sn9—Tm1^ii^	168.76 (6)
Sn7^vi^—Tm3—Sn6	139.03 (3)	Sn9^ii^—Sn9—Tm1^ii^	70.64 (3)
Sn7^vii^—Tm3—Sn6	76.34 (2)	Sn9^xiv^—Sn9—Tm1^ii^	70.64 (3)
Ni11—Tm3—Sn6^i^	111.10 (4)	Ni10—Sn9—Sn7^xi^	50.37 (3)
Ni11^i^—Tm3—Sn6^i^	49.56 (3)	Sn9^ii^—Sn9—Sn7^xi^	165.45 (5)
Sn7^vi^—Tm3—Sn6^i^	76.34 (2)	Sn9^xiv^—Sn9—Sn7^xi^	88.68 (2)
Sn7^vii^—Tm3—Sn6^i^	139.03 (3)	Tm1^ii^—Sn9—Sn7^xi^	123.61 (3)
Sn6—Tm3—Sn6^i^	88.65 (3)	Ni10—Sn9—Sn7	50.37 (3)
Ni11—Tm3—Sn4^v^	47.77 (3)	Sn9^ii^—Sn9—Sn7	88.68 (2)
Ni11^i^—Tm3—Sn4^v^	47.77 (3)	Sn9^xiv^—Sn9—Sn7	165.45 (5)
Sn7^vi^—Tm3—Sn4^v^	128.566 (19)	Tm1^ii^—Sn9—Sn7	123.61 (3)
Sn7^vii^—Tm3—Sn4^v^	128.566 (19)	Sn7^xi^—Sn9—Sn7	85.04 (3)
Sn6—Tm3—Sn4^v^	87.74 (2)	Ni10—Sn9—Tm3^iii^	68.89 (5)
Sn6^i^—Tm3—Sn4^v^	87.74 (2)	Sn9^ii^—Sn9—Tm3^iii^	129.93 (3)
Ni11—Tm3—Sn8	126.12 (3)	Sn9^xiv^—Sn9—Tm3^iii^	129.93 (3)
Ni11^i^—Tm3—Sn8	126.12 (3)	Tm1^ii^—Sn9—Tm3^iii^	99.87 (3)
Sn7^vi^—Tm3—Sn8	60.46 (2)	Sn7^xi^—Sn9—Tm3^iii^	54.95 (2)
Sn7^vii^—Tm3—Sn8	60.46 (2)	Sn7—Sn9—Tm3^iii^	54.95 (2)
Sn6—Tm3—Sn8	79.58 (2)	Ni10—Sn9—Tm1	60.61 (3)
Sn6^i^—Tm3—Sn8	79.58 (2)	Sn9^ii^—Sn9—Tm1	56.25 (3)
Sn4^v^—Tm3—Sn8	162.20 (3)	Sn9^xiv^—Sn9—Tm1	110.83 (5)
Ni11—Tm3—Sn5^viii^	47.97 (4)	Tm1^ii^—Sn9—Tm1	126.90 (2)
Ni11^i^—Tm3—Sn5^viii^	105.23 (3)	Sn7^xi^—Sn9—Tm1	109.43 (3)
Sn7^vi^—Tm3—Sn5^viii^	122.60 (3)	Sn7—Sn9—Tm1	59.51 (2)
Sn7^vii^—Tm3—Sn5^viii^	67.83 (2)	Tm3^iii^—Sn9—Tm1	113.14 (2)
Sn6—Tm3—Sn5^viii^	88.266 (18)	Ni10—Sn9—Tm1^xi^	60.61 (3)
Sn6^i^—Tm3—Sn5^viii^	150.70 (3)	Sn9^ii^—Sn9—Tm1^xi^	110.83 (5)
Sn4^v^—Tm3—Sn5^viii^	63.03 (2)	Sn9^xiv^—Sn9—Tm1^xi^	56.25 (3)
Sn8—Tm3—Sn5^viii^	128.29 (2)	Tm1^ii^—Sn9—Tm1^xi^	126.90 (2)
Ni11—Tm3—Sn5^ix^	105.23 (3)	Sn7^xi^—Sn9—Tm1^xi^	59.51 (2)
Ni11^i^—Tm3—Sn5^ix^	47.97 (4)	Sn7—Sn9—Tm1^xi^	109.43 (3)
Sn7^vi^—Tm3—Sn5^ix^	67.83 (2)	Tm3^iii^—Sn9—Tm1^xi^	113.14 (2)
Sn7^vii^—Tm3—Sn5^ix^	122.60 (3)	Tm1—Sn9—Tm1^xi^	76.84 (2)
Sn6—Tm3—Sn5^ix^	150.70 (3)	Sn9—Ni10—Sn7^xi^	79.56 (5)
Sn6^i^—Tm3—Sn5^ix^	88.266 (18)	Sn9—Ni10—Sn7	79.56 (5)
Sn4^v^—Tm3—Sn5^ix^	63.03 (2)	Sn7^xi^—Ni10—Sn7	119.29 (8)
Sn8—Tm3—Sn5^ix^	128.29 (2)	Sn9—Ni10—Sn6	124.03 (8)
Sn5^viii^—Tm3—Sn5^ix^	80.44 (2)	Sn7^xi^—Ni10—Sn6	119.49 (4)
Ni11—Tm3—Sn9^vii^	111.84 (4)	Sn7—Ni10—Sn6	119.49 (4)
Ni11^i^—Tm3—Sn9^vii^	111.84 (4)	Sn9—Ni10—Sn8^iii^	132.26 (8)
Sn7^vi^—Tm3—Sn9^vii^	59.24 (2)	Sn7^xi^—Ni10—Sn8^iii^	76.87 (5)
Sn7^vii^—Tm3—Sn9^vii^	59.24 (2)	Sn7—Ni10—Sn8^iii^	76.87 (5)
Sn6—Tm3—Sn9^vii^	133.565 (16)	Sn6—Ni10—Sn8^iii^	103.71 (7)
Sn6^i^—Tm3—Sn9^vii^	133.565 (16)	Sn9—Ni10—Tm1	75.45 (4)
Sn4^v^—Tm3—Sn9^vii^	108.72 (3)	Sn7^xi^—Ni10—Tm1	150.48 (7)
Sn8—Tm3—Sn9^vii^	89.08 (3)	Sn7—Ni10—Tm1	71.26 (3)
Sn5^viii^—Tm3—Sn9^vii^	64.13 (2)	Sn6—Ni10—Tm1	65.06 (4)
Sn5^ix^—Tm3—Sn9^vii^	64.13 (2)	Sn8^iii^—Ni10—Tm1	132.15 (3)
Ni11—Tm3—Ni10^vii^	132.07 (3)	Sn9—Ni10—Tm1^xi^	75.45 (4)
Ni11^i^—Tm3—Ni10^vii^	132.07 (3)	Sn7^xi^—Ni10—Tm1^xi^	71.26 (3)
Sn7^vi^—Tm3—Ni10^vii^	45.186 (14)	Sn7—Ni10—Tm1^xi^	150.48 (7)
Sn7^vii^—Tm3—Ni10^vii^	45.186 (14)	Sn6—Ni10—Tm1^xi^	65.06 (4)
Sn6—Tm3—Ni10^vii^	111.93 (3)	Sn8^iii^—Ni10—Tm1^xi^	132.15 (3)
Sn6^i^—Tm3—Ni10^vii^	111.93 (3)	Tm1—Ni10—Tm1^xi^	87.31 (5)
Sn4^v^—Tm3—Ni10^vii^	151.69 (4)	Sn9—Ni10—Tm2^xi^	137.40 (2)
Sn8—Tm3—Ni10^vii^	46.11 (3)	Sn7^xi^—Ni10—Tm2^xi^	65.00 (3)
Sn5^viii^—Tm3—Ni10^vii^	96.20 (3)	Sn7—Ni10—Tm2^xi^	137.87 (7)
Sn5^ix^—Tm3—Ni10^vii^	96.20 (3)	Sn6—Ni10—Tm2^xi^	62.75 (4)
Sn9^vii^—Tm3—Ni10^vii^	42.97 (3)	Sn8^iii^—Ni10—Tm2^xi^	62.99 (4)
Ni11^x^—Sn4—Ni11^v^	120.64 (7)	Tm1—Ni10—Tm2^xi^	127.82 (6)
Ni11^x^—Sn4—Ni11	103.86 (4)	Tm1^xi^—Ni10—Tm2^xi^	71.45 (2)
Ni11^v^—Sn4—Ni11	103.86 (4)	Sn9—Ni10—Tm2	137.40 (2)
Ni11^x^—Sn4—Sn8^iii^	109.77 (4)	Sn7^xi^—Ni10—Tm2	137.87 (7)
Ni11^v^—Sn4—Sn8^iii^	109.77 (4)	Sn7—Ni10—Tm2	65.00 (3)
Ni11—Sn4—Sn8^iii^	107.99 (5)	Sn6—Ni10—Tm2	62.75 (4)
Ni11^x^—Sn4—Tm2	164.54 (4)	Sn8^iii^—Ni10—Tm2	62.99 (4)
Ni11^v^—Sn4—Tm2	74.78 (3)	Tm1—Ni10—Tm2	71.45 (2)
Ni11—Sn4—Tm2	69.01 (3)	Tm1^xi^—Ni10—Tm2	127.82 (6)
Sn8^iii^—Sn4—Tm2	61.91 (2)	Tm2^xi^—Ni10—Tm2	84.84 (4)
Ni11^x^—Sn4—Tm2^xi^	74.78 (3)	Sn9—Ni10—Tm3^iii^	68.13 (5)
Ni11^v^—Sn4—Tm2^xi^	164.54 (4)	Sn7^xi^—Ni10—Tm3^iii^	59.65 (4)
Ni11—Sn4—Tm2^xi^	69.01 (3)	Sn7—Ni10—Tm3^iii^	59.65 (4)
Sn8^iii^—Sn4—Tm2^xi^	61.91 (2)	Sn6—Ni10—Tm3^iii^	167.84 (7)
Tm2—Sn4—Tm2^xi^	89.78 (3)	Sn8^iii^—Ni10—Tm3^iii^	64.13 (4)
Ni11^x^—Sn4—Tm2^v^	70.41 (4)	Tm1—Ni10—Tm3^iii^	122.41 (4)
Ni11^v^—Sn4—Tm2^v^	70.41 (4)	Tm1^xi^—Ni10—Tm3^iii^	122.41 (4)
Ni11—Sn4—Tm2^v^	71.55 (4)	Tm2^xi^—Ni10—Tm3^iii^	109.00 (4)
Sn8^iii^—Sn4—Tm2^v^	179.53 (4)	Tm2—Ni10—Tm3^iii^	109.00 (4)
Tm2—Sn4—Tm2^v^	117.81 (2)	Sn4^x^—Ni11—Sn4^v^	120.64 (7)
Tm2^xi^—Sn4—Tm2^v^	117.81 (2)	Sn4^x^—Ni11—Sn6	117.99 (4)
Ni11^x^—Sn4—Tm3^v^	63.03 (4)	Sn4^v^—Ni11—Sn6	117.99 (4)
Ni11^v^—Sn4—Tm3^v^	63.03 (4)	Sn4^x^—Ni11—Sn5^viii^	83.76 (5)
Ni11—Sn4—Tm3^v^	142.59 (5)	Sn4^v^—Ni11—Sn5^viii^	83.76 (5)
Sn8^iii^—Sn4—Tm3^v^	109.43 (3)	Sn6—Ni11—Sn5^viii^	121.24 (7)
Tm2—Sn4—Tm3^v^	130.985 (18)	Sn4^x^—Ni11—Sn4	76.14 (4)
Tm2^xi^—Sn4—Tm3^v^	130.985 (18)	Sn4^v^—Ni11—Sn4	76.14 (4)
Tm2^v^—Sn4—Tm3^v^	71.04 (2)	Sn6—Ni11—Sn4	100.37 (6)
Ni11^x^—Sn4—Sn4^v^	126.78 (6)	Sn5^viii^—Ni11—Sn4	138.39 (7)
Ni11^v^—Sn4—Sn4^v^	54.61 (4)	Sn4^x^—Ni11—Tm3	154.05 (7)
Ni11—Sn4—Sn4^v^	49.25 (3)	Sn4^v^—Ni11—Tm3	69.20 (2)
Sn8^iii^—Sn4—Sn4^v^	121.54 (3)	Sn6—Ni11—Tm3	67.24 (4)
Tm2—Sn4—Sn4^v^	59.64 (2)	Sn5^viii^—Ni11—Tm3	73.14 (4)
Tm2^xi^—Sn4—Sn4^v^	116.83 (4)	Sn4—Ni11—Tm3	129.35 (3)
Tm2^v^—Sn4—Sn4^v^	58.17 (3)	Sn4^x^—Ni11—Tm3^xi^	69.20 (2)
Tm3^v^—Sn4—Sn4^v^	108.24 (3)	Sn4^v^—Ni11—Tm3^xi^	154.05 (7)
Ni11^x^—Sn4—Sn4^x^	54.61 (4)	Sn6—Ni11—Tm3^xi^	67.24 (4)
Ni11^v^—Sn4—Sn4^x^	126.78 (6)	Sn5^viii^—Ni11—Tm3^xi^	73.14 (4)
Ni11—Sn4—Sn4^x^	49.25 (3)	Sn4—Ni11—Tm3^xi^	129.35 (3)
Sn8^iii^—Sn4—Sn4^x^	121.54 (3)	Tm3—Ni11—Tm3^xi^	92.41 (5)
Tm2—Sn4—Sn4^x^	116.83 (4)	Sn4^x^—Ni11—Tm2	135.01 (7)
Tm2^xi^—Sn4—Sn4^x^	59.64 (2)	Sn4^v^—Ni11—Tm2	63.75 (3)
Tm2^v^—Sn4—Sn4^x^	58.17 (3)	Sn6—Ni11—Tm2	61.53 (3)
Tm3^v^—Sn4—Sn4^x^	108.24 (3)	Sn5^viii^—Ni11—Tm2	137.92 (2)
Sn4^v^—Sn4—Sn4^x^	85.36 (4)	Sn4—Ni11—Tm2	61.07 (3)
Ni12—Sn5—Ni11^xii^	118.18 (7)	Tm3—Ni11—Tm2	70.75 (2)
Ni12—Sn5—Tm3^xii^	137.69 (2)	Tm3^xi^—Ni11—Tm2	128.71 (6)
Ni11^xii^—Sn5—Tm3^xii^	58.89 (3)	Sn4^x^—Ni11—Tm2^xi^	63.75 (3)
Ni12—Sn5—Tm3^xiii^	137.69 (2)	Sn4^v^—Ni11—Tm2^xi^	135.01 (7)
Ni11^xii^—Sn5—Tm3^xiii^	58.89 (3)	Sn6—Ni11—Tm2^xi^	61.53 (3)
Tm3^xii^—Sn5—Tm3^xiii^	80.44 (2)	Sn5^viii^—Ni11—Tm2^xi^	137.92 (2)
Ni12—Sn5—Tm1^xi^	59.57 (4)	Sn4—Ni11—Tm2^xi^	61.07 (3)
Ni11^xii^—Sn5—Tm1^xi^	138.857 (19)	Tm3—Ni11—Tm2^xi^	128.71 (6)
Tm3^xii^—Sn5—Tm1^xi^	153.58 (3)	Tm3^xi^—Ni11—Tm2^xi^	70.75 (2)
Tm3^xiii^—Sn5—Tm1^xi^	94.310 (14)	Tm2—Ni11—Tm2^xi^	82.84 (4)
Ni12—Sn5—Tm1	59.57 (4)	Sn4^x^—Ni11—Tm2^v^	60.33 (4)
Ni11^xii^—Sn5—Tm1	138.857 (19)	Sn4^v^—Ni11—Tm2^v^	60.33 (4)
Tm3^xii^—Sn5—Tm1	94.310 (14)	Sn6—Ni11—Tm2^v^	160.53 (7)
Tm3^xiii^—Sn5—Tm1	153.58 (3)	Sn5^viii^—Ni11—Tm2^v^	78.23 (5)
Tm1^xi^—Sn5—Tm1	78.88 (2)	Sn4—Ni11—Tm2^v^	60.16 (4)
Ni12—Sn6—Ni10	111.40 (7)	Tm3—Ni11—Tm2^v^	123.78 (4)
Ni12—Sn6—Ni11	126.13 (7)	Tm3^xi^—Ni11—Tm2^v^	123.78 (4)
Ni10—Sn6—Ni11	122.47 (7)	Tm2—Ni11—Tm2^v^	104.85 (4)
Ni12—Sn6—Tm2^xi^	134.083 (17)	Tm2^xi^—Ni11—Tm2^v^	104.85 (4)
Ni10—Sn6—Tm2^xi^	69.57 (3)	Sn8—Ni12—Sn8^xi^	126.20 (9)
Ni11—Sn6—Tm2^xi^	70.88 (3)	Sn8—Ni12—Sn6	113.33 (5)
Ni12—Sn6—Tm2	134.083 (17)	Sn8^xi^—Ni12—Sn6	113.33 (5)
Ni10—Sn6—Tm2	69.57 (3)	Sn8—Ni12—Sn5	87.74 (6)
Ni11—Sn6—Tm2	70.88 (3)	Sn8^xi^—Ni12—Sn5	87.74 (6)
Tm2^xi^—Sn6—Tm2	90.64 (3)	Sn6—Ni12—Sn5	123.90 (9)
Ni12—Sn6—Tm1	65.63 (4)	Sn8—Ni12—Sn7^vii^	75.93 (5)
Ni10—Sn6—Tm1	66.95 (3)	Sn8^xi^—Ni12—Sn7^vii^	75.93 (5)
Ni11—Sn6—Tm1	135.524 (14)	Sn6—Ni12—Sn7^vii^	93.61 (7)
Tm2^xi^—Sn6—Tm1	136.53 (3)	Sn5—Ni12—Sn7^vii^	142.49 (8)
Tm2—Sn6—Tm1	74.304 (16)	Sn8—Ni12—Tm1^xi^	156.15 (8)
Ni12—Sn6—Tm1^xi^	65.63 (4)	Sn8^xi^—Ni12—Tm1^xi^	69.53 (3)
Ni10—Sn6—Tm1^xi^	66.95 (3)	Sn6—Ni12—Tm1^xi^	67.18 (4)
Ni11—Sn6—Tm1^xi^	135.524 (14)	Sn5—Ni12—Tm1^xi^	74.18 (5)
Tm2^xi^—Sn6—Tm1^xi^	74.304 (16)	Sn7^vii^—Ni12—Tm1^xi^	127.76 (4)
Tm2—Sn6—Tm1^xi^	136.53 (3)	Sn8—Ni12—Tm1	69.53 (3)
Tm1—Sn6—Tm1^xi^	88.94 (3)	Sn8^xi^—Ni12—Tm1	156.15 (8)
Ni12—Sn6—Tm3^xi^	79.46 (4)	Sn6—Ni12—Tm1	67.18 (4)
Ni10—Sn6—Tm3^xi^	135.290 (16)	Sn5—Ni12—Tm1	74.18 (5)
Ni11—Sn6—Tm3^xi^	63.20 (3)	Sn7^vii^—Ni12—Tm1	127.76 (4)
Tm2^xi^—Sn6—Tm3^xi^	72.611 (17)	Tm1^xi^—Ni12—Tm1	90.29 (6)
Tm2—Sn6—Tm3^xi^	134.01 (3)	Sn8—Ni12—Tm2^vii^	63.18 (5)
Tm1—Sn6—Tm3^xi^	144.75 (4)	Sn8^xi^—Ni12—Tm2^vii^	63.18 (5)
Tm1^xi^—Sn6—Tm3^xi^	80.659 (16)	Sn6—Ni12—Tm2^vii^	155.17 (8)
Ni12—Sn6—Tm3	79.46 (4)	Sn5—Ni12—Tm2^vii^	80.93 (6)
Ni10—Sn6—Tm3	135.290 (16)	Sn7^vii^—Ni12—Tm2^vii^	61.56 (4)
Ni11—Sn6—Tm3	63.20 (3)	Tm1^xi^—Ni12—Tm2^vii^	126.83 (4)
Tm2^xi^—Sn6—Tm3	134.01 (3)	Tm1—Ni12—Tm2^vii^	126.83 (4)

Symmetry codes: (i) *x*, *y*+1, *z*; (ii) −*x*+1, −*y*, −*z*; (iii) −*x*+1/2, −*y*, *z*−1/2; (iv) −*x*+1/2, −*y*+1, *z*−1/2; (v) −*x*, −*y*, −*z*; (vi) −*x*+1/2, −*y*+1, *z*+1/2; (vii) −*x*+1/2, −*y*, *z*+1/2; (viii) *x*−1/2, *y*, −*z*+1/2; (ix) *x*−1/2, *y*+1, −*z*+1/2; (x) −*x*, −*y*−1, −*z*; (xi) *x*, *y*−1, *z*; (xii) *x*+1/2, *y*, −*z*+1/2; (xiii) *x*+1/2, *y*−1, −*z*+1/2; (xiv) −*x*+1, −*y*−1, −*z*.
